# Clinical features which predict neuronal surface autoantibodies in new-onset focal epilepsy: implications for immunotherapies

**DOI:** 10.1136/jnnp-2020-325011

**Published:** 2020-11-20

**Authors:** Ronan N McGinty, Adam Handel, Teresa Moloney, Archana Ramesh, Andrew Fower, Emma Torzillo, Holger Kramer, Stephen Howell, Patrick Waters, Jane Adcock, Arjune Sen, Bethan Lang, Sarosh R Irani

**Affiliations:** 1 Oxford Autoimmune Neurology Group, Nuffield Department of Clinical Neurosciences, University of Oxford, Oxford, UK; 2 Department of Neurology, John Radcliffe Hospital, Oxford University Hospitals, Oxford, UK; 3 MRC London Institute of Medical Sciences, Imperial College, London, UK; 4 Department of Neurology, Sheffield Teaching Hospitals NHS Foundation Trust, The University of Sheffield, Sheffield, UK

**Keywords:** autoimmune encephalitis, epilepsy, neuroimmunology

## Abstract

**Objective:**

To generate a score which clinically identifies surface-directed autoantibodies in adults with new-onset focal epilepsy, and evaluate the value of immunotherapy in this clinical setting.

**Methods:**

Prospective clinical and autoantibody evaluations in a cohort of 219 consecutive patients with new-onset focal epilepsy.

**Results:**

10.5% (23/219) of people with new-onset focal epilepsy had detectable serum autoantibodies to known or novel cell surface antigenic targets. 9/23 with autoantibodies were diagnosed with encephalitis, by contrast to 0/196 without autoantibodies (p<0.0001). Multivariate analysis identified six features which predicted autoantibody positivity (area under the curve=0.83): age ≥54 years, ictal piloerection, lowered self-reported mood, reduced attention, MRI limbic system changes and the absence of conventional epilepsy risk factors. 11/14 (79%) patients with detectable autoantibodies, but without encephalitis, showed excellent long-term outcomes (modified Rankin Score=0) despite no immunotherapy. These outcomes were superior to those of immunotherapy-treated patients with confirmed autoantibody-mediated encephalitis (p<0.05).

**Conclusions:**

Seizure semiology, cognitive and mood phenotypes, alongside inflammatory investigation findings, aid the identification of surface autoantibodies among unselected people with new-onset focal epilepsy. The excellent immunotherapy-independent outcomes of autoantibody-positive patients without encephalitis suggests immunotherapy administration should be guided by clinical features of encephalitis, rather than autoantibody positivity. Our findings suggest that, in this cohort, immunotherapy-responsive seizure syndromes with autoantibodies largely fall under the umbrella of autoimmune encephalitis.

## Introduction

Neuronal surface-directed antibodies (NSAbs) are considered pathogenic in patients with autoimmune encephalitis (AE). AE commonly presents with prominent seizures and neuropsychiatric features and shows a preferential response to immunotherapies versus anti-seizure medications (ASMs).^1–4^ This has prompted the introduction of ‘epilepsy of immune aetiology’ within the International League Against Epilepsy (ILAE) 2017 classification.^5^ The same NSAbs, as well as high levels of antibodies to intraneuronal glutamic acid decarboxylase-65 (GAD65), are also described in the serum of people with more isolated forms of epilepsy, without core features of encephalitis.^6–8^ In this context, their clinical, aetiological and therapeutic relevance is unclear, but of major potential importance to all neurologists who manage new-onset epilepsy. In our large, prospective, real-world study of new-onset focal epilepsy, we predicted that *formes frustes* of AE would help identify clinical features suggesting the presence of NSAbs and asked whether detection of these NSAbs should alter patient management.

## Materials and methods

Between 9 December 2011 and 4 November 2015, consecutive adult patients (≥18 years) with a diagnosis of new-onset focal epilepsy and their first seizure within the previous 12 months were prospectively recruited from the routine practice of two epileptologists at the Oxford University Hospitals NHS Foundation Trust. Written informed consent and sera were obtained (Ethical approvals: Oxfordshire RECA 07/Q160X/28 and REC16/YH/0013). Clinical data gathered at onset (online supplemental table 1) included detailed phenotype and investigation results, Quality of Life in Epilepsy-31, Hospital Anxiety and Depression Score, Addenbrooke’s Cognitive Examination (ACE) and modified Rankin Score (mRS); as well as information to inform the Antibody Prevalence in Epilepsy and Encephalopathy (APE2) score (online supplemental table 2)^9 10^ and diagnostic criteria for possible or definite AE.^11^ Subsequently, 1-year and 3-year mRS were ascertained from patients with NSAbs.

10.1136/jnnp-2020-325011.supp1 Supplementary data

For NSAbs, sera were tested against autoantigen-expressing live HEK293 cells (live cell-based assay; online supplemental table 3), and for reactivity with the surface of live cultured hippocampal neurons, using sensitive protocols.^12 13^ Autoantibodies to GAD65 were determined using a commercial radioimmunoprecipitation assay.

Statistical analysis was conducted in R (V.3.6.1). Dimensionality reduction was performed using Multiple Factor Analysis in ‘FactoMineR’ with up to 10% missing data imputed using missForest. Stepwise Bayesian general linear modelling analysis was undertaken using ‘arm’. Wilson 95% CIs with continuity correction were calculated using ‘DescTools’.

## Results

### NSAb findings

Of 241 recruited patients, 22 were excluded (online supplemental table 4). Of the remaining 219, median age was 49 years (range 16–91) and 109 (49.8%) were female. In 23/219 (10.5%) patients, serum NSAbs were detected across candidate and novel autoantigens (table 1) including roughly equal frequencies against leucine-rich glioma inactivated-1 (LGI1), contactin-associated protein-like 2 (CASPR2), plus the *N*-methyl-d-aspartate receptor (NMDAR) and γ-aminobutyric acid _A/B_ receptors (GABA_A_R and GABA_B_R). An additional five patients had antibodies to the surface of live neurons, without an established autoantigen. Autoantibodies to contactin-2, the glycine receptor and the α-amino-3-hydroxy-5-methyl-4-isoxazole propionic acid receptor (AMPAR) were each found in one patient. No dipeptidyl-peptidase-like protein 6 (DPPX) or high-titre GAD65 antibodies were detected. Overall, from the 23 people with NSAbs, 9 had a clinical diagnosis of AE (7/9 fulfilling published criteria).^11^ By contrast, none of the 196 without NSAbs had a clinical diagnosis of AE (p<0.0001; Fisher’s exact test).

**Table 1:** Clinical and laboratory features of patients with epilepsy and positive neuronal surface autoantibodies

Age	Sex	Autoantibody	Live hippocampal neuron binding	APE2 score	Criteria AE	Clinical AE	Pilo	Epil RF	Lowered Mood	ACE Att	MRI Δlimbic	Number of ASMs	mRS 1 year	mRS 3 years	Immunotherapy
79	M	CASPR2	+	3	Definite	Yes	No	None	No	14	Yes	2	3	2	IVMP, Pred, IVIg, PLEX
55	F	CASPR2		6	Definite	Yes	No	None	Yes	18	Yes	3	2	2	IVMP, Pred, IVIg, PLEX
55	F	GABA_B_R	+	9	Definite	Yes	No	None	No	NA	Yes	6	6	NA	IVMP, Pred, PLEX
18	F	NMDAR	+	8	Definite	Yes	No	None	Yes	15	Yes	3	2	2	IVMP, Pred, PLEX, Aza, MMF
60	F	NMDAR		4	Definite	Yes	No	None	Yes	13	No	3	0	0	Pred
77	F	GABA_A_R, GABA_B_R	+	8	Possible	Yes	No	None	Yes	18	Yes	4	1	0	Pred
54	M	LGI1	+	6	Possible	Yes	Yes	None	Yes	18	Yes	2	0	0	None
59	F	LGI1	+	4	No	Yes	No	None	Yes	18	No	1	1	0	None
69	M	Unknown	+	4	No	Yes	No	None	No	13	No	1	1	2	Pred, Aza
63	M	AMPAR	+	2	No	No	No	None	No	NA	No	1	0	0	None
28	M	CASPR2		2	No	No	No	FHx	Yes	17	No	1	0	0	None
41	F	CASPR2		2	No	No	No	None	Yes	18	No	0	0	0	None
75	F	Contactin-2		1	No	No	No	None	No	NA	No	1	0	0	None
37	F	GABA_A_R		2	No	No	No	None	Yes	18	No	2	0	0	None
36	F	GABA_B_R		2	No	No	No	None	No	18	No	1	1	0	None
69	F	GlyR		4	No	No	No	None	Yes	15	No	4	1	1	None
61	M	LGI1	+	2	No	No	Yes	None	Yes	18	No	1	0	0	None
36	F	NMDAR		2	No	No	No	None	Yes	18	No	1	2	2	None
68	F	NMDAR, GABA_A_R		1	No	No	No	None	No	18	No	1	0	0	None
77	F	Unknown	+	2	No	No	No	None	Yes	NA	No	0	0	NA	None
55	F	Unknown	+	4	No	No	No	None	Yes	18	No	5	2	2	None
91	M	Unknown	+	1	No	No	No	None	No	9	No	1	6	NA	None
37	M	Unknown	+	2	No	No	No	None	Yes	18	No	2	0	0	None

APE2 score of 4 or more is considered positive. Diagnosis of encephalitis based on clinical impression (‘Clinical AE’; corresponding rows shaded in grey) or published criteria (‘Criteria AE’), as per Graus *et al*.^11^

‘Unknown’ autoantibody specificities reflect IgG binding to the surface of live hippocampal neurons, without a known antigenic target.

ACE Att, Addenbrooke’s Cognitive Examination attention domain score; AE, autoimmune encephalitis; APE2, Antibody Prevalence in Epilepsy and Encephalopathy Score; ASM, anti-seizure medication; Aza, azathioprine; Epil RF, epilepsy risk factors (neonatal, perinatal, focal neurological insults, family history of epilepsy or Alzheimer’s disease); FHx, family history; IVIg, intravenous immunoglobulin; IVMP, intravenous methylprednisolone; mRS, modified Rankin Score; Myco, mycophenolate; NA, not available; PLEX, plasma exchange; Pred, prednisolone.

### Factors associated with the presence of NSAbs and AE

**Figure 1:**
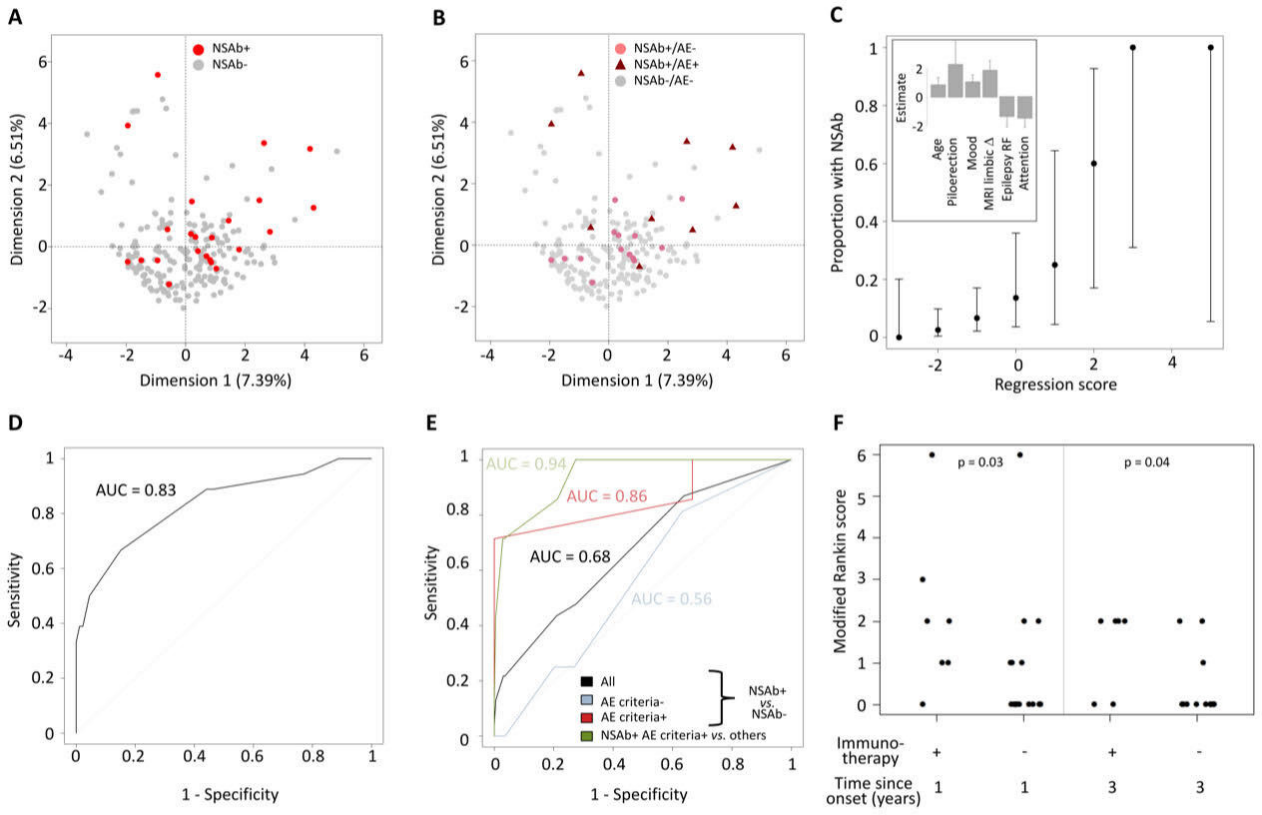
Clinical phenotypes associated with NSAb status in new-onset focal epilepsy. The first two dimensions are shown, highlighting: (A) NSAb-positive (red) or NSAb-negative (grey) status and (B) NSAb-positive (pale red) or NSAb-negative (grey) without encephalitis (dots), or NSAb-positive (dark red) with clinically diagnosed autoimmune encephalitis (triangles). (C) The proportion of patients by total model score. Error bars show 95% CIs. The inset shows the weighting and SE of each factor within the regression model. (D) Receiver operator characteristic (ROC) curve of the total model score for predicting NSAb status across all patients. (E) ROC curve of the APE2 score for predicting NSAb status across all patients (black), patients not meeting the criteria for autoimmune encephalitis (blue), patients meeting the criteria for autoimmune encephalitis (red) and predicting NSAb-positive criteria-confirmed autoimmune encephalitis across all patients. (F) Scatter plot of modified Rankin score in NSAb-positive patients by immunotherapy status over time (Mann-Whitney U test p values<0.05). AE, autoimmune encephalitis; APE2, Antibody Prevalence in Epilepsy and Encephalopathy; epilepsy RF, epilepsy risk factors; MRI limbic Δ, changes within the limbic system on MRI.

Dimensionality reduction with multiple factor analysis showed that patients were highly heterogeneous and the modest clustering of those with NSAbs was largely driven by a clinical diagnosis of AE (figure 1A, B). Univariate analysis identified 11 clinical parameters that differed significantly between patients with and without NSAbs: age (p=0.04), ictal piloerection (p=0.02), lesional MRI (p=0.04), self-reported mood disturbance (p=0.007), ACE attention domain (p=0.01), ACE total score (p=0.04), QOLIE-31 score (p=0.02), self-reported neuropsychiatric features (p=0.03), epilepsy risk factors (p=0.05), inflammatory cerebrospinal fluid (CSF; p=0.004) and limbic system lesions on MRI (p=0.0002). A multivariate stepwise regression model allocated weighted scores to six of these: age ≥54 years=+1, self-reported mood disturbance=+1, limbic system lesions on MRI=+2, ictal piloerection=+2.5, ACE attention score ≥16=−1.5 and epilepsy risk factors=−1.5 (figure 1C). The probability of NSAb positivity increased with higher scores (Spearman’s ρ=0.99, p<0.0001; figure 1C) and receiver operating characteristic (ROC) analysis confirmed these features strongly predicted NSAb status (area under the curve (AUC)=0.83; total score ≥0; sensitivity=66.7%, specificity=84.9%; figure 1D). By contrast, the APE2 score performed less well in predicting NSAb status (sensitivity 43.5%, specificity 79.1%, AUC=0.68) and more accurately predicted criteria-defined AE, particularly if associated with NSAbs (sensitivity 85.7%, specificity 78.8%, AUC=0.94; figure 1E).

### Comparisons of those with and without AE

From 23 patients with NSAbs (table 1), a comparison of those with (n=9) and without (n=14) a clinical diagnosis of AE revealed several differences in the AE cohort: more ASMs (median of 3 vs 1; p=0.0073), more frequent immunotherapies (7/9 vs 0/14, p=0.0001), higher APE2 scores (median of 6 vs 2; p<0.0001), more frequent MRI limbic inflammation (6/9 vs 0/14; p=0.0008) and a trend towards greater positivity of serum IgGs targeting the surface of live neurons (7/9 vs 5/14, p=0.09). Compared with the seven patients administered immunotherapy, those with NSAbs who were not administered immunotherapy showed lower disability after 1 and 3 years (both p<0.05), and 11/16 (68.8%) were asymptomatic at 3-year follow-up (mRS=0; figure 1F). Hence, despite no immunotherapy, patients with NSAbs, but without AE, generally showed good outcomes.

## Discussion

In this prospective study of 219 consecutive adults with new-onset focal epilepsy, NSAb status was best predicted by a combination of clinical parameters which closely resemble features observed in AE. Almost half of our patients with NSAbs were diagnosed with AE, and ~30% fulfilled stringent criteria for AE.^11^ Of those with NSAbs and more isolated forms of epilepsy, without individual features of AE, almost all were treated with ASMs alone and typically remained asymptomatic at long-term follow-up. Overall, these findings suggest that detection of NSAbs in patients with new-onset seizures, but without features of AE, should not alter current clinical management. Our observations should help guide the frequent clinical dilemma of which patients with new-onset seizures to test for autoantibodies and subsequently treat with immunotherapy. Taken together, our data suggest the clinical phenotype is paramount in guiding the relevance of autoantibody results, and provide data to address an outstanding question from a recent ILAE consensus statement.^7^

This ILAE statement also highlighted controversy over the term ‘autoimmune epilepsy’.^7^ In routine clinical practice, this nomenclature acts as a valuable signpost and aide memoire when seeing patients with seizures.^2 14^ However, ‘epilepsy’ carries several social stigmata and is defined by an enduring tendency to seizures. In AE, this lifelong risk is refuted by a recent study,^4^ despite several forms of AE commonly leading to hippocampal atrophy.^2–4 7 10^ The alternative concept of acute symptomatic seizures may more accurately capture the nature of seizures in patients with AE. Data-driven modifications to nomenclature will benefit from longer-term follow-up studies.

Ictal piloerection, low mood and attention and MRI limbic system changes are recognised features of late-onset AE, particularly in association with LGI1 antibodies.^2 4 14 15^ The absence of movement disorders or more diffuse cognitive impairment as predictive factors in our model suggests the overall syndrome may reflect a *formes frustes* of AE. This contrasts with APE2 score parameters,^9^ which appear to largely reflect more florid features seen in classical AE.

Our observational study has several limitations. These include limited CSF autoantibody measurements, which reflected UK practice particularly at the start of the study period. Yet, w ithout this valuable parameter, a diagnosis of NMDAR-antibody encephalitis is still possible.^11^ Yet, two of our four patients with serum NMDAR antibodies did not have features consistent with encephalitis, likely suggesting detection of clinically unrelated serum antibodies in these cases. In addition, our series in total only identified nine AE cases, although this may be considered substantial given the largely outpatient-based recruitment. This, and the high (~10%) seroprevalence rate, may reflect a referral bias given Oxford’s interest in AE, but is well aligned with other available estimates.^6 9 10^ Our serological data identified some samples with NSAbs proven by live cell-based assays, but without concomitant cell surface neuronal reactivities. This was especially evident in the cohort without a clinical diagnosis of AE, and perhaps these antibodies reflect low-affinity or low-titre autoantibodies which are not disease relevant. Their specificity, however, remains reassuring given their typical selectivity for just one of eight surface-expressed autoantigens.

In the future, our prediction model will benefit from validation in independent, larger studies which may compare the risk of enduring seizures in the NSAb-positive versus NSAb-negative populations, with and without AE, something which we did not survey at follow-up. Hence, we cannot comment on long-term seizure status in the 5/16 patients (31%) who had NSAbs, no diagnosis of AE and 3-year mRS >0. In these patients, it remains possible that immunotherapy would have led to a greater benefit. However, in our view, this finding is more likely to be consistent with the predicted ~30% of all people with epilepsy who are known to become ASM resistant: this provides a testable hypothesis for a future randomised controlled trial.

Overall, our observations support the concept that, in patients who present with new-onset focal seizures, clinical features which are consistent with a ‘mild encephalitis’ helps identify those with NSAbs which should alter patient management. This clinico-serological syndrome appeared characteristic and its recognition will improve detection and treatment of these patients. These findings should discourage widespread screening strategies to identify patients with autoantibodies among unselected seizure cohorts.
