# Supplementary material for: Clinical features which predict neuronal surface autoantibodies in new-onset focal epilepsy: implications for immunotherapies
Source: J Neurol Neurosurg Psychiatry. 2020 Nov 20;92(3):291–4. doi: 10.1136/jnnp-2020-325011 (PMC7892387; doi:10.1136/jnnp-2020-325011)
Supplement: Supplementary data [file jnnp-2020-325011supp001.pdf]

Supplementary Table 1. Information captured by clinical phenotyping database

Clinical features
Sex
Antibody status
Date of birth
Date of study recruitment
Date of first seizure
Epilepsy risk factors (neonatal, perinatal, focal neurological insults, family history of epilepsy or Alzheimer's disease)
Formal psychiatric disorder prior to epilepsy diagnosis
Personal or family history of autoimmunity
Personal history of malignancy
History of status epilepticus
Self-reported neuropsychiatric changes (memory/psychosis/mood/anxiety)<br>Number of seizures in 2/6/12/24/36/48 months from onset
Total number, type and duration of anti-seizure medications (ASMs) trialled
Non-ASM medications including immunotherapy trials for seizure-control (date/type/route/course length/dose)
Number of seizures in first 3/6/12/24/36/48 months after ASM commenced
Seizure-cessation reached (Yes/No)
Time to seizure-cessation from starting ASM (months)
Adverse effect from ASM
Investigations
Serum sodium
MRI result
EEG result
Clinical assessments/scores
Hospital Anxiety and Depression Score (HADS)
Addenbrooke's Cognitive Examination-Revised (ACE-R) score
Quality of Life in Epilepsy-31 (QOLIE-31) score
Antibody Prevalence in Epilepsy and Encephalopathy (APE2) score
Autoimmune encephalitis (clinical diagnosis and as defined by Graus et al[11])

Supplementary Table 2. Antibody Prevalence in Epilepsy and Encephalopathy Score (APE2) criteria. The maximum score is 18 points, and a total score of 4 or more has been considered suggestive for underlying autoantibodies.[10]

**Criteria**	**Points**
New-onset, rapidly progressive mental status changes or new-onset seizure activity	**+1**
Neuropsychiatric changes	**+1**
Autonomic dysfunction	**+1**
Viral prodrome	**+2**
Faciobrachial dystonic movements	**+3**
Facial dyskinesias without faciobrachial dystonic movements	**+2**
Seizures refractory to at least two anti-seizure medicines	**+2**
CSF inflammation	**+2**
Brain MRI consistent with encephalitis	**+2**
Systemic malignancy within five years of neurological symptom onset	**+2**

Supplementary Table 3. Autoantibody detection. Live cell-based assays (CBA) were used to detect NSAbs to leucine-rich glioma inactivated-1 (LGI1), contactin-associated protein-like 2 (CASPR2), contactin-2, dipeptidyl-peptidase-like protein 6 (DPPX), N-methyl-D-aspartate receptor (NMDA-R), α-amino-3-hydroxy-5-methyl-4-isoxazole propionic acid receptor (AMPA-R), γ-aminobutyric acid A receptor ($GABA_AR$), γ-aminobutyric acid B receptor ($GABA_BR$) and the glycine receptor. A radioimmunoprecipitation assay (RIPA) was used to detect antibodies to glutamic acid decarboxylase-65 (GAD65). Live neuronal-based assays (NBA) tested for antibody binding to neuronal surface antigens.

Target antigen	Assay method
GAD65	RIPA
LGI1	Live CBA
CASPR2	Live CBA
Contactin-2	Live CBA
AMPA-R	Live CBA
$GABA_AR$	Live CBA
$GABA_BR$	Live CBA
Glycine receptor	Live CBA
NMDA-R	Live CBA
Hippocampal neuron surface binding without the above reactivities	Live NBA

Supplementary Table 4. Patients excluded from study

**Reason for exclusion**	**Number of patients**
<18 years	1
Duplicate registration	1
Generalised epilepsy	3
Epilepsy diagnosis >12months	5
Focal epilepsy diagnosis unconfirmed	9
Clinical information unavailable	1
No serum samples received	2
